# Nonpigmented Metastatic Melanoma in a Two-Year-Old Girl: A Serious Diagnostic Dilemma

**DOI:** 10.1155/2015/298273

**Published:** 2015-02-11

**Authors:** Gulden Diniz, Hulya Tosun Yildirim, Selcen Yamaci, Nur Olgun

**Affiliations:** ^1^Izmir Tepecik Education and Research Hospital, Pathology Laboratory, Turkey; ^2^Izmir Dr. Behcet Uz Children's Hospital, Pathology Laboratory and Dermatology Clinics, Turkey; ^3^Pediatric Oncology Clinics, Izmir Dokuz Eylul University, Turkey

## Abstract

Although rare, malignant melanoma may occur in children. Childhood melanomas account for only 0.3–3% of all melanomas. In particular the presence of congenital melanocytic nevi is associated with an increased risk of development of melanoma. We herein report a case of malignant melanoma that developed on a giant congenital melanocytic nevus and made a metastasis to the subcutaneous tissue of neck in a two-year-old girl. The patient was hospitalized for differential diagnosis and treatment of cervical mass with a suspicion of hematological malignancy, because the malignant transformation of congenital nevus was not noticed before. In this case, we found out a nonpigmented malignant tumor of pleomorphic cells after the microscopic examination of subcutaneous lesion. Nonpigmented metastatic melanoma was diagnosed by several immunohistochemical and flow cytometric studies. She was offered palliative chemotherapy; however, her parents did not accept treatment. The patient died within 9 months of diagnosis. We emphasized here that the possibility of malignant melanoma in the differential diagnosis of childhood tumors should be kept in mind.

## 1. Introduction

Malignant melanoma in children and adolescence is rare. Childhood melanoma accounts for less than 3% of all malignancies seen in children under the age of 18 years [1–3]. The presence of congenital melanocytic nevi depending on the size of the lesions is one of the risk factors for developing childhood melanoma because of the possible malignant transformation [3]. Childhood melanoma is a potentially fatal disease, and it is important to remember MM is a differential diagnosis of any pigmented or also nonpigmented lesion in a child [1–4].

Congenital melanocytic nevi (CMN) are present at birth or appear during the first weeks of life [5]. CMN occur in approximately 1% of newborns and are usually classified according to their size. The New York University Registry defines 3 groups according to the largest diameter of the projected adult size: small (less than 1.5 cm), medium (1.5 to 19.9 cm), and large or giant (greater than 20 cm). And also, the facial nevi greater than 1% of the body surface area or body nevi greater than 2% of the body surface area can be accepted as giant CMN (GCMN). Prevalence of GCMN has been rarely specified. Estimated prevalence varies widely, from 1 : 20.000 to 1 : 500.000 [5–8]. The vast majority of CMN are of small size with very low potential malignancy. The giant lesions are 200 times less frequent than small ones, can carry psychosocial problems, and increased risks for MM [5]. Also, it is well known that neurogenic sarcoma, liposarcoma, rhabdomyosarcoma, and undifferentiated small round cell tumors can also be formed on GCMN [5–10].

The occurrence of MM of the scalp and its metastasis is extremely rare in early childhood. We report a case of amelanotic nodular MM with metastasis originating from GCMN of the scalp. In addition, the clinicopathological features of this patient and the main points of differential diagnosis are discussed.

## 2. Case Presentation

A 2-year-old girl was referred for the presence of a subcutaneous palpable mass on the neck. She appeared otherwise healthy with no evidence of lymphadenopathy or organomegaly. Her parents were consanguineously married. She had no significant family history of atypical nevus or melanoma. She has a giant congenital nevus on head but nobody has not noticed that the malignant melanoma nodules were developed on it.

**Figure 1:**
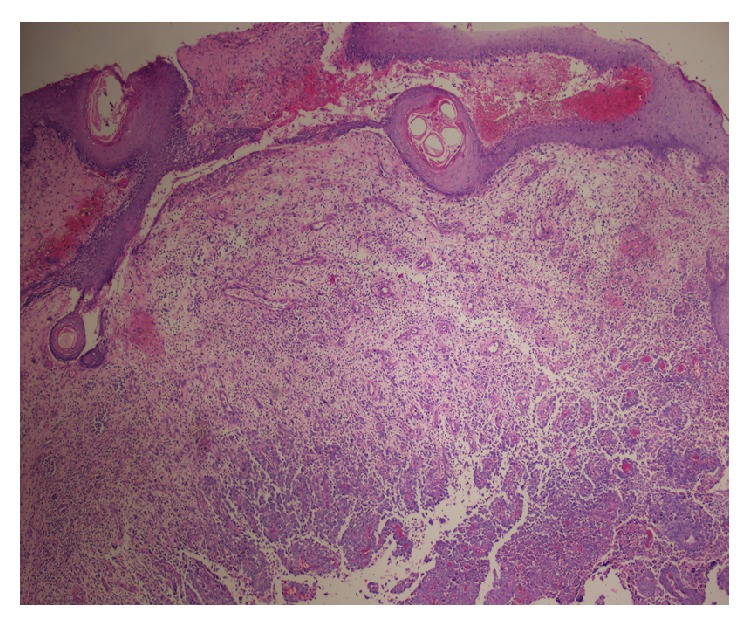
Ulcerated metastatic melanoma with solid growth pattern (HE ×40).

**Figure 2:**
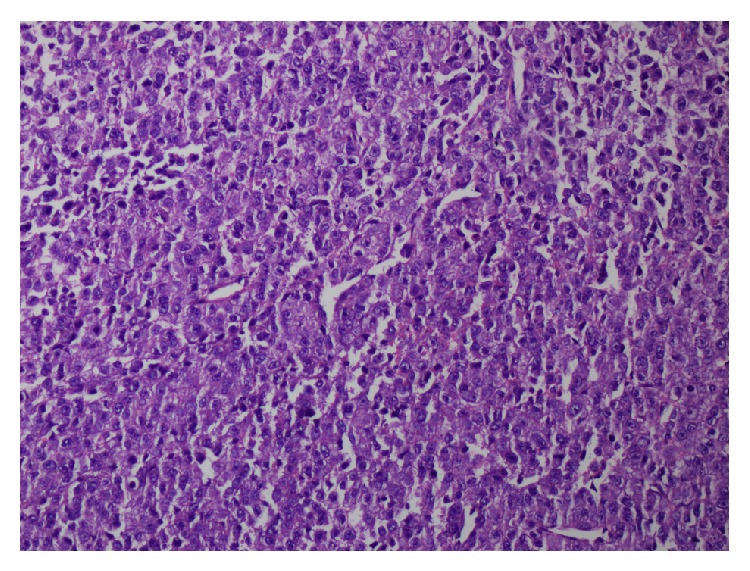
Tumor cells composed of large epithelioid cells with no melanin pigment (HE ×200).

**Figure 3:**
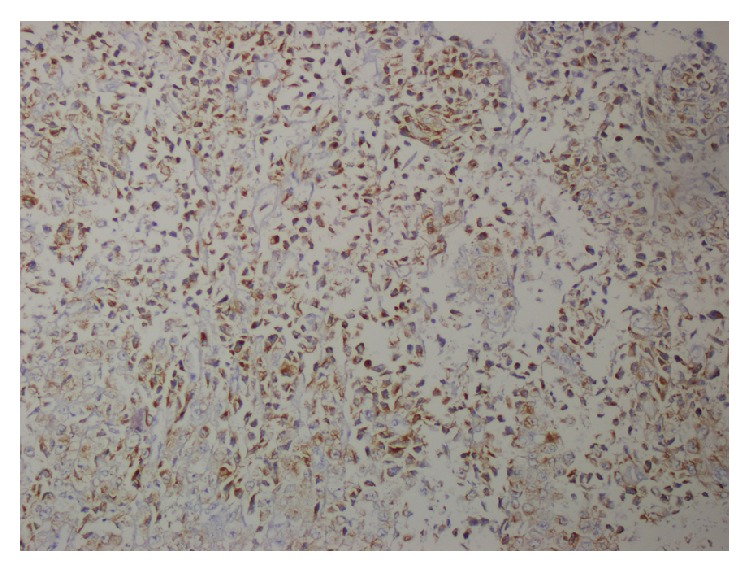
Immunohistochemically, HMB-45 positive tumor cells (DAB ×200).

On physical examination, on the left sternocleidomastoid region, indurated subcutaneous palpable masses were adjacent to each other and adhered to the skin with erythema formation. These indurations were palpated as solid masses with patchy areas of softening. Initially two biopsy specimens measuring 5 and 6 mm in diameter were excised from this subcutaneous palpable mass. Histopathological examination revealed a highly malignant tumor diffusely infiltrating the soft tissue (Figure 1). Tumor cells composed of large epithelioid cells arranged in a solid and infiltrative pattern which comprised of atypical tumor cells with abundant mitotic cells (Figure 2). Tumor cells had enlarged hyperchromatic nuclei with prominent nucleoli devoid of melanin pigment. In immunohistochemical studies, tumor cells revealed positive expressions for HMB-45 (Figure 3), S-100, and Melan A (MART1) but negative for myeloperoxidase, LCA, pancytokeratin, CD 68, desmin, and CD56. With these findings metastatic malignant melanoma was diagnosed.

The requested dermatological examination revealed a large congenital melanocytic nevus completely covering the left half of the scalp, and extending to the left side of the neck and showing heterogeneous dark hyperpigmentation with several nodules measuring 0.5–1 cm in diameter. With these clinical findings, development of malignant melanoma on congenital melanocytic nevus and also soft tissue metastasis was suspected and biopsy of nevus was taken. Microscopically, the similar histopathological morphology was seen. The histology revealed ulcerated amelanotic nodular melanoma composed of medium-large sized malignant cells exhibiting atypical vesicular nuclei and prominent nucleoli but no melanin pigment. The tumor infiltrated into the entire dermis up to the hypodermis and the surgical margin was positive. Numerous and atypical mitotic cells were detected. Immunohistochemically, tumor cells showed reactivity for Melan A (MART1), S100, and HMB-45. Total body contrast-enhanced computed tomography (CT) examination did not reveal any further secondary localizations and metastases. Skin-grafting or total excision was not made because of lesion's size and localization. The final diagnosis was ulcerated metastatic amelanotic nodular malignant melanoma with a congenital melanocytic nevus and the patient was transferred to the department of internal medicine for chemotherapy. However, her family refused additional treatment and she died 9 months later after the diagnosis.

## 3. Discussion

Congenital melanocytic nevi occur in approximately 1% of newborns and are defined as benign nevomelanocytic proliferations. They are present at birth or appear during the first weeks of life [5, 7]. They may have a heritable component and can present with marked differences in size, shape, color, and location. CMN can exhibit distinctive histologic features that can help in differentiating them from other nevi especially from common acquired nevi. In neonates they have often junctional pattern. However they may also show compound, or dermal nevus patterns. The nevomelanocytes in CMN tend to extend deeper into the dermis and subcutaneous tissues to be aligned between collagen bundles and to track along skin appendages and neurovascular structures [5, 8]. In the present case, the main component of the tumor was present as ulcerated amelanotic nodular MM. However on the lateral side of the tumor, nevomelanocytic lesions with a junctional component which tended to extend into subcutaneous tissue with positive surgical margins were observed.

GCMN developed* in utero* are present approximately 1 in 20.000 to in 50.000 newborns. These nevi develop often between the 9th and 20th week of gestation. Nevus cells are derived from neural crest melanocytes [6]. Alikhan et al. [9] reported that melanocytes migrate through the dorsolateral pathway between dermatomes to the overlying ectoderm. Migration stops when the melanocytes reach the epidermis. Eventually, melanocytes produce melanin pigment, sometimes in excessive amounts. In our case, the giant congenital melanocytic nevus contained excessive amounts of melanin pigment, but there was no pigment in malignant melanoma.

The lifetime risk of malignancy for patients with GCMN ranges from 0 to 42%. The risk of melanoma formation depends strongly on the size of CMN, with a lifetime risk of 3.1% for GCMN [9]. In our case GCMN was completely covering the left half of the scalp, extending to the left neck. Therefore, the lesion could not be completely excised because of the anatomical location and size of the GCMN.

MM is usually readily diagnosed by the presence of melanin granules and its growth pattern. Although amelanotic MM contains no melanin granules, it is often difficult to differentiate it from lymphoma, sarcoma, or epithelial tumors. Immunohistochemical staining must be performed to establish the diagnosis [11]. Immunohistochemical staining with HMB-45 is one of the most useful marker for the diagnosis of amelanotic MM. The majority of pediatric malignant melanomas arise* de novo. *A significant portion (up to 50%) of pediatric melanomas are amelanotic and show a nodular configuration (up to 30%) compared with adult melanomas [1–4]. In our case, nodular malignant melanoma had not melanin pigment and showed nodular configuration with surgical margin positivity.

Cutaneous MM differs in children in that it may arise from conditions unique to the pediatric population. Because of this, pediatric melanoma is classified by the mode of occurrence as transplacental melanoma which transmitted from the mother to the fetus in utero, as transformation from giant congenital melanocytic nevus, as association with congenital predisposing conditions such as xeroderma pigmentosum and dysplastic nevus syndrome, albinism, or as development from healthy skin [3–5]. In our case, MM was transformed from GCMN. Even most patients with GCMN will not develop melanoma but they may have increased risks of MM and other malignancies. It is generally accepted that a complete excision of the lesion in the early childhood decreases the risk of malignancy [1–4].

In this report, we describe a rare case of GCMN with metastatic ulcerated amelanotic melanoma in a 2-year-old child, in consideration of its clinical, histological, and immunohistochemical features. Amelanotic nodular MM is difficult to differentiate from lymphoma, other epithelial and nonepithelial malignancies due to absence of melanin pigment. It can be easily mistaken for other malignancies both clinically and pathologically. We believe that such rare cases must be reported in the literature, so that sufficient data will accumulate in time, which may clarify the disease process and guide correct management of the affected children in the future.

In conclusion, if a malignant melanoma has not melanin pigment and its primary site is not known, then it is difficult to make an accurate and specific diagnosis. Since it is an aggressive tumor, the importance of early diagnosis is unquestionable. We emphasize here to be aware of the fact that the patient's story and physical examination are the most essential steps of pathological diagnosis of a nonpigmented malignant melanoma metastasis in a child.
